# Density-Based Entropy Centrality for Community Detection in Complex Networks

**DOI:** 10.3390/e25081196

**Published:** 2023-08-11

**Authors:** Krista Rizman Žalik, Mitja Žalik

**Affiliations:** Faculty of Electrical Engineering and Computer Science, University of Maribor, 2000 Maribor, Slovenia

**Keywords:** networks, undirected graphs, community detection, node centrality, label propagation

## Abstract

One of the most important problems in complex networks is the location of nodes that are essential or play a main role in the network. Nodes with main local roles are the centers of real communities. Communities are sets of nodes of complex networks and are densely connected internally. Choosing the right nodes as seeds of the communities is crucial in determining real communities. We propose a new centrality measure named density-based entropy centrality for the local identification of the most important nodes. It measures the entropy of the sum of the sizes of the maximal cliques to which each node and its neighbor nodes belong. The proposed centrality is a local measure for explaining the local influence of each node, which provides an efficient way to locally identify the most important nodes and for community detection because communities are local structures. It can be computed independently for individual vertices, for large networks, and for not well-specified networks. The use of the proposed density-based entropy centrality for community seed selection and community detection outperforms other centrality measures.

## 1. Introduction

Complex networks represent complex interactions among multiple nodes representing objects in many real systems. One of the most important problems in complex networks is the location of nodes that are essential or play a main role within the network. Nodes having main local roles are the centers of real communities.

Communities are sets of nodes joined together in tightly connected groups, with only a few connections with nodes belonging to other communities. They can be used as an intermediate step to select the most influential nodes. By incorporating information on the community structure of the input network into the optimization process of influence maximization, the efficiency of the most influential nodes is improved [1].

An analysis of complex networks can uncover new knowledge and improve our understanding of the processes and structures of networks [2]. One important aspect of network analysis is to uncover the community structure, which has been shown to be an important property of networks [3].

Many community-detection methods [4] have been proposed, and some of them can also cope efficiently with dynamic networks [5]. Global community-detection methods require the structural information of the whole networks, while local community-detection algorithms require only the local information of one subnetwork. Many existing global community-detection approaches use a network’s most important nodes, i.e., seeds, and some global scoring functions in the optimizing process for seed identification and the expansive growth of each seed into a larger set of similar nodes named communities [6]. Many local community-detection algorithms also start from a number of the locally most important nodes (seeds) and expand them into communities by examining only the neighborhood of the seeds. The chosen seed nodes have a great influence on the identified communities and on the performance and efficiency of the community-detection methods [7,8]. Until now, different centrality measures tied to the network topology have been introduced to solve the issue of finding these initial community seeds, as the locally most influential nodes in a complex network [9].

Several centrality measures take advantage of various network topological properties to estimate the influence. However, the majority of centrality measures ignore the community structure, although it is one of the main features of many real-world networks. The centrality of a node in a network depends on two influences: its local influence on the neighboring nodes belonging to its community and its global influence on the nodes belonging to the other communities. The goal of our research was to create a new centrality measure, which can enable better estimation of the local importance and identification of the locally most important nodes. They are the centers of real communities. The new centrality measure can be used for the ranking nodes regarding the node’s importance in one network, and it is not appropriate for a comparison of the importance of values from different complex networks. The important nodes from different complex networks can have different centrality values, while the complex networks may not have the same structure. Centrality measures have to provide a quick prediction of real communities in large complex networks for the task of community detection. Therefore, an efficient centrality measure should not require processing of a whole network but require only local information of the subnetwork. The new local centrality measure should also be based on empirical observations rather than on a theoretical analysis, so that it can be used in real systems to identify the most important nodes as the centers of the communities in a network. The realization of this goal has practical value, since the nodes with the highest centrality values can be used directly for the identification of real communities. On the other hand, our research also has a theoretical value: achieving an understanding of the ability to identify communities using the new proposed factor density-based entropy, which can be estimated by humans or calculated automatically.

We introduce a local entropy-based centrality measure that can enable better identification of the most important central vertices as the seeds of real communities in networks. Furthermore, the proposed entropy-based centrality measure requires no control parameters to be tuned to achieve the optimal solution. The entropy-based centrality measure used considers the nodes and the number of links in the neighborhood of a node.

In this work, we make the following contributions. We extend the current list of the significant factors described in previous research on node centrality with entropy. We demonstrate that the proposed density-based centrality measure is correlated with the local centrality and correlated weakly with the global nodes’ centrality measures. This makes it useful for the identification of the locally most important nodes in a network, which are the seeds of the communities, while the communities are local structures.

We use an extended simple label propagation community-detection algorithm, LPA [10], with different centrality measures for the identification of communities and prove the efficiency of the proposed centrality measure. We extended the LPA using the seed nodes and link strength to create cores of the communities around the identified seed nodes before detecting whole communities, since the recent research has shown that maximal neighbor similarity reveals real communities [11].

We show that the proposed density-based entropy centrality outperforms the other local centrality measures in identifying real community structures. The proposed density-based entropy centrality measure identifies all the centers that are related to the centers of natural communities. The proposed centrality and extended LPA community-detection method enable local community detection. The density-based entropy centrality is a local node centrality index, and the link strength used in the extended LPA algorithm for community identification is a local similarity index. They enable local community identification of any number of communities, while no prior knowledge is required about the number of communities.

The rest of this article is organized as follows. Section 2 presents related work. Section 3 gives basic definitions and proposes the density-based entropy centrality. Section 4 proposes the use of the centrality for community detection with the extended LPA. Section 5 provides the experiments and results. Finally, Section 6 concludes this article.

## 2. Related Work

Each centrality measure considers some of the properties of complex systems and establishes its own definition of centrality, while each community-detection method considers some of the properties of the complex systems and establishes its own definition of a community [12]. We introduce a local entropy-based centrality measure that can enable better identification of the most important central vertices as the seeds of real communities of networks.

The modern science of networks has brought significant advances to our understanding of complex systems and different community-detection methods [13].

Many community-detection algorithms select the seed vertices randomly to detect communities using methods that enhance or expand the k-means clustering method [14]. The label propagation methods take all vertices as possible seeds of communities at the beginning [10]. Weskida [15] showed the use of an evolutionary algorithm for selecting the seeds in social networks. Erlandsson et al. [16] identified the most influential users using association rule learning. Gleich and Seshadhri [17] proposed a localized method of detecting seeds, which are vertices with local minimal conductance. However, most existing local community-detection algorithms use one centrality index to identify the most important vertices as the seeds for communities and then extend the seeds into communities by running a greedy optimization process using one quality function [12].

Finding influential nodes in a complex network is an important research topic [18,19]. Different centrality measures tied to the network topology have been introduced for finding these influential nodes [20]. Several centrality measures take advantage of various network topological properties to evaluate the influence, while the majority of research works ignore the network community structure, although it is one of the main features of real-world networks.

## 3. Centrality Measures

Node centrality is one of the most intuitive methods to identify the important nodes in a network. Node centrality evaluates the efficiency of the propagation of information from a central node and estimates the influence and the strength of the connection of the node with its neighborhood [21].

The popular centrality measures are degree centrality [22,23], betweenness centrality [24,25], and closeness centrality [26]. A significant correlation between node degree and the geometric centrality measures and also between other centrality measures has been reported in [27]. The betweenness centrality and closeness centrality belong to the global-based approaches which exploit the information of the whole network to rank nodes with their topological importance in the network.

Let $G_{U}$ be an input undirected unweighted graph consisting of a set of vertices *V* and a set of edges E⊂V×V. *A* is an adjacency matrix, sometimes also called the connection matrix, with rows and columns labeled with the graph vertices, with values $A_{i,j}=1$ when the vertices *i* and *j* are adjacent (connected with an edge) or 0 otherwise.

Degree centrality finds the most connected vertex with the greatest degree as the most central. The degree centrality $D_{i}$ of a vertex *i* is proportional to the degree (or number of directly connected neighbors) of that vertex.

(1) $$D_{i}=\frac{\sum_{j!=i,j=1}^{n}A_{ij}}{n-1}$$

Closeness centrality finds the vertex with the smallest distances to all other vertices in the network as the most central.

(2) $$C_{i}=\frac{n-1}{\sum_{j!=i,j=1}^{n}d\left(i,j\right)}$$

where d(i,j) is the shortest path distance between the vertices *i* and *j*.

Betweenness centrality identifies the vertex of the graph with the highest number of shortest paths going through the vertex as the most central.

(3) $$B_{i}=\sum_{i!=j!=k}^{}\frac{\sigma_{jk}\left(i\right)}{\sigma_{jk}}$$

where $\sigma_{jk}$ is the total number of shortest paths from the graph vertex *j* to *k*, and $\sigma_{jk}\left(i\right)$ is the number of those paths from vertex *j* to *k* that pass through graph vertex *i*. The use of betweenness and closeness centrality makes the algorithm costly because they use the whole network for evaluation of the centrality of each vertex, while the degree centrality is a local centrality measure. Studies in [28] showed that the degree-based and centrality-based approaches may result in less influence over the network because these measures do not consider the effect of the neighborhood.

Other proposed centrality measures are the eigencentrality measure [29,30], information centrality [31], and communicability centrality [32].

The eigencentrality makes the centrality of a graph vertex proportional to the sum of the centralities of its neighborhood.

(4) $$c_{i}=\frac{1}{\lambda_{max}}\sum_{j!=i,j=1}^{n}A_{ij}c_{j};i=1,2\ldots n$$

where $\lambda_{max}$ is the largest eigenvalue of the adjacency matrix *A*.

Google’s PageRank [33] and the Katz [34] centrality are variants of the eigenvector centrality.

The information centrality observes how information flows between all the pairs of vertices in the network.

(5) $$I_{i}=\frac{n}{\sum_{j=1}^{n}I_{ij}}$$

$I_{ij}$ is the combined path information. It can be computed from the matrix D(i,j) containing the number of links that share the paths in a combined path.

The communicability centrality [32] is a subgraph centrality. It is calculated from all the closed paths of all lengths that start and end at a graph vertex *i*. Paths with a shorter length have a greater influence on the centrality of the vertex *i*. The communicability of a vertex *i* is calculated using the exponential of the adjacency matrix *A*:

(6) $$Comm\left(i\right)={\left[e^{A}\right]}_{ii}$$

Vertices with more neighbors have a greater influence on their surroundings than vertices with few ties with their neighborhood and can propagate information to the other vertices in the network more efficiently. Because they have many links with the surrounding vertices, they are often involved in exchanges with other vertices. They have access to more resources of the network. Therefore, a node degree is a very simple and effective local centrality measure.

Degree centrality calculates the centrality using only direct neighbors, but those neighbors can be disconnected from the whole network, and therefore, it can identify the local unimportant centers. However, local metrics like degree centrality are relatively simple and less effective in the identification of the central vertices of a whole network, although the global metrics, such as closeness and betweenness centralities, can identify the most important vertices of the whole network better. Nevertheless, the local metrics of centrality are more efficient in revealing real community centers, while the communities are local structures. The extent to which different centrality measures offer unique or redundant information depends on the topology of the network. Past empirical work has identified correlations between the different centrality measures in different applications. As an example, the closeness and eigenvector centralities were correlated very highly in a network of collaborations between high-energy physicists (r = 0.91), but not in a Power Grid network (r = −0.04) [35]. The different centrality measures identify different choices of the most central vertex within a graph. The centrality which is optimal for one application can be sub-optimal for a different application. As an example, individuals who influence the flow around a system have the greatest betweenness centrality, while the betweenness centrality is not efficient for community detection, where the graph vertices with the highest betweenness centrality can be bridges between two or more communities or the central vertices of communities. The closeness centrality identifies the nodes which influence the entire network most quickly. The degree centrality can best identify locally popular or informed individuals, which can be the centers of real communities. They are sometimes bridges between two or more communities and not the centers of real communities. Therefore, we want to define a centrality measure that can identify the centers of real communities better.

## 4. Density-Based Entropy Centrality

We exploited the entropy and cliques in the proposed centrality measure. The cliques are ideal communities’ structures, which are subsets of individuals who interact with each other more frequently than other individuals outside the clique. This is a similar definition to the definition of a community. A clique represents a densely connected structure in a graph, and, as such, it can be used to recover the locally most related elements useful for several data mining tasks such as clustering, frequent patterns, and community mining [36]. The cliques can also be used in the optimization functions of community detection. In [20], a novel community-detection method was proposed that minimizes a new objective function, called the clique conductance.

Mhadhbi et al. [37] solved the problem of influence maximization using a maximal clique problem. Their solution is based on the fact that the presence of a dense neighborhood around a network node is fundamental to the maximization of the influence.

We built our centrality measure from the following simple and relevant principle: a node that is a good infector can be contained in multiple cliques. A dense neighborhood around a node maximizes the influence and spread of the information.

Network nodes with the maximal proposed centrality should contain the most information presented in the network. The Shannon entropy is related to the information present in systems. In the research on complex networks, a number of different entropy measures have been introduced [38,39], where the entropy is used to analyze the statistical behavior or the structural features of a network.

### 4.1. Basic Definitions

Let $G_{U}$ be an input undirected unweighted graph consisting of a set of vertices $V=v_{1},\ldots ,v_{n}$ and a set of *m* edges E⊂V×V that models a network with *n* nodes and *m* links. $A(v_{i},v_{j})$ is an adjacency matrix, sometimes also called a connection matrix with rows and columns labeled by the graph vertices, with a value of 1 when the vertices are adjacent (connected with an edge) or 0 otherwise.

Using a weight function, we obtained a weighted graph *G* from unweighted $G_{U}$. *G* is an undirected weighted graph defined with an ordered triplet G(V,E,ω), where the third element of the triplet is a weight function $\omega :V\times V\to R^{+}⋃0$ satisfying ω(u,v)=ω(v,u) for all u,v∈V. The weighted adjacency matrix *W* of the graph is defined as W(i,j)=ω(vi,vj). Since *G* is an undirected graph, we have $W=W^{T}$. We used the weighted function ω, which estimates all the maximal cliques to which an edge belongs.

A clique is a subset of the vertices in a graph, also called complete subgraphs, where all the vertices are adjacent to each other. A k-clique is a complete subgraph consisting of *k* vertices all with pairwise connections, where *k* is any positive integer. A maximal clique is a clique that cannot be extended by including one more adjacent graph vertex, meaning it is not a subset of a larger clique.

Clique and maximal clique are defined below.

Let G=(V,E) be an undirected graph. Then, a clique *C* of graph *G* is a subset of the vertices C⊆V such that whenever v1 and v2 belong to clique *C*, then the edge (v1,v2) belongs to *E*.

A clique *C* of *G* is maximal if, for any $x\in V\setminus C,C⋃\subseteq \left\{x\right\}$ is not a clique. A maximal clique is a clique that cannot be extended by including one more adjacent graph vertices.

The number of vertices constituting a clique δ is called the size of the clique and is denoted as ϑ(δ).

In this paper, we use $S_{k}$ to represent the collection of all maximal k-cliques and $S={⋃}_{k}^{}S_{k}$ to represent the collection of all maximal cliques.

We introduce a new weighted graph *G*, which contains the maximal-clique information of the unweighted graph $G_{U}$. G=(V,E,ω) is an undirected weighted graph, where the weight function $\omega (v_{i},v_{j})$ is the sum of the sizes $\vartheta (\delta_{max})$ of the maximal cliques $\delta_{max}$ that the graph vertex $v_{i}$ and vertex $v_{j}$ both engage with. ω measures how densely two vertices are connected.

(7) $$\omega \left(v_{i},v_{j}\right)=\sum_{\delta \in S}^{}\;\sum_{v_{i},v_{j}\in \delta }^{}\vartheta \left(\delta \right)$$

### 4.2. Density-Based Entropy Centrality

By requiring only the sum of information of the local maximal cliques, we encode all the clique information adaptively and obtain a computationally inexpensive measure in comparison with global measures. Links between the vertices in a graph that belong to the more maximal cliques are more important. The importance of links is measured with link strength. The link strength of an edge between vertices $v_{i}$ and $v_{j}$ is 1 when the vertices do not belong to any maximal clique, and $1+\omega (v_{i},v_{j})$ otherwise:

(8) $$W\left(v_{i},v_{j}\right)=\left(1+\omega \left(v_{i},v_{j}\right)\right)\cdot A\left(v_{i},v_{j}\right)$$

Link strength is a localized vertices similarity index for assessing the similarity between adjacent vertices. A larger link strength value means a stronger relationship between two adjacent vertices. The calculation of link strength between adjacent vertices involves the cliques containing both the adjacent vertices. To denote that two connected vertices are more similar, although they do not belong to any clique, than two not connected vertices, we increased the weight value ω by 1.

We used the entropy of the link strength of all the graph vertex neighbors to calculate the density-based entropy centrality. The density-based entropy centrality $C_{E}$ of a graph vertex *v* is

(9) $$C_{E}\left(v\right)=-\sum_{v_{i}\in N\left(v\right)}\frac{W(v,v_{i})}{\sum_{v_{j}\in N\left(v\right)}W\left(v,v_{j}\right)}\cdot log\frac{W(v,v_{i})}{\sum_{v_{j}\in N\left(v\right)}W\left(v,v_{j}\right)}$$

where N(v) is the neighbors of the graph vertex *v*:

(10) $$N\left(v_{i}\right)=\left\{v_{i}|\left(v,v_{i}\right)\in E\right\}$$

where $\left(v,v_{i}\right)$ is a link and *E* is the set of links of the graph. The proposed density-based centrality reveals the locally most important vertices, which are at the center of a denser subgraph compared with their surroundings.

We defined the sum of weights of the edges, which connect a graph vertex with its neighbor vertices, as clique centrality. The clique centrality $C_{C}$ of a vertex *v* is

(11) $$C_{C}\left(v\right)=\sum_{v_{i}\in N\left(v\right)}W(v,v_{i})$$

Clique centrality finds the graph vertex with the greatest sum of link strengths as the most important, although it is not at the center of a dense subgraph.

## 5. Evaluating the Efficiency of Density-Based Entropy Centrality for Community Detection

The centrality value of each network node can be calculated using the proposed density-based centrality. All the network nodes with centrality values greater than their neighbors are the locally most important nodes. They can be used as seeds in any community-detection method and the weights of network links determined via link strength can also be used in any community-detection method.

For evaluation of the proposed centrality measure, we extended and used the label propagation algorithm (LPA) because the LPA is a simple and fast community-detection algorithm with a nearly linear time complexity [10]. Instead of selecting network nodes randomly for label propagation used in LPA, we used the identified nodes with the highest local values of density-based entropy centrality as the seeds of communities. Then, communities were created using label propagation.

We first calculated the cliques, the link strength for each link, and the density-based entropy for each network node. Then, we detected the seeds and formed the cores of the communities. We finished with community extension step using the LPA algorithm (see Algorithm 1).    
**Algorithm 1:** CDCE. **Data**: graph $G_{U}\left(V,E\right)$ with a set of vertices *V*, and a set of edges *E* **Result**: C is a set of core vertices Identify all max cliques Calculate link strength $W(\left[v_{i}\right]\left[v_{j}\right]$ (Equation (8)) for all edges. **for** *all $v_{i}\in V$***:**(   Calculate $C_{E}\left[v_{i}\right].$ (Equation (9)) Sort vertices by importance. Seed and core detection (G(V,E,W)). Community extension-label propagation algorithm-LPA (G(V,E,W)).

The following three steps are necessary in the extended LPA method for community detection using a density-based entropy centrality named CDCE.

Step 1: Calculate the density-based entropy centrality. First, we have to calculate the influence power with the proposed density-based entropy centrality (Equation (9)).

Step 2: Identify the seeds of communities and the cores of communities. The vertices are labeled using sequential integer values. The vertices in the center of the density subgraphs on the density peaks have a higher density-based entropy centrality than the others. The vertices with a higher density-based entropy centrality than all their neighbor vertices are seeds. The seed node labels become the community labels. We have a seed *s* and assign a seed’s neighbor vertex *i* to the same community as the seed if it has all neighbor vertices with a smaller value of the density-based entropy centrality than vertex *i* and seed *s*. Such vertices form, together with seed node, the core of community (see Algorithm 2). The neighbor node of seed node *s* connected with the greatest link strength *maxLinkStrength* among all links with the seed’s neighbors becomes a member of the core of community *s*. All the neighbor vertices of node *i* connected with a link strength greater than 0.9 · *maxLinkStrength* also form the same core of community *i*. Some networks, like the football network described in the next Section, consist of vertices with the same or nearly the same degrees (number of neighbor vertices). In such networks, there are a lot of core vertices.

Step 3: Identify communities using the LPA algorithm.
**Algorithm 2:** Core detection. **Data**: Graph G(V,E,W) with a node set *V*, edge set *E* and link strength matrix     $W\left[v_{i}\right]\left[v_{j}\right]$ **Result**: Vector commNo[v] with set of id of community to which each node *v*
     belongs, and a vector containing core vertices core[v]; **for** *all v∈V***:**   commNo[v] = −1; **for** *all v∈V***:**   **for** *all $v_{i}\in N\left(v\right)$*
**:**    search the neighbor maxSim with max link strength $W\left[v\right]\left[v_{i}\right]$   greater = 1;
**for** *all $v_{i}\in N\left(v\right)$*
**:**    **if** *!*(*$C_{E}\left[v\right]\geq C_{E}\left[v_{i}\right]$ or
*(*$C_{E}\left[v\right]<C_{E}\left[v_{i}\right]$ and*     *$W\left[v_{i}\right]\left[v\right]>W\left[v_{i}\right]\left[v_{k}\right]\forall v_{k}\in N\left(v_{i}-v\right)))$*
**:**      greater = 0;   **if** *greater* = 1**:**    core[v] = 1; commNo[v] = v;
**if** *comm[maxSim]=−1***:**      core[maxSim]=1;commNo[maxSim]=commNo[v];    **else:**      core[v]=1;commNo[v]=commNo[maxSim];    **for** *all $v_{i}\in N\left(v\right)$*
**:**      **if** *$LinkStrength\left[v\right]\left[v_{i}\right]>0.9\cdot maxLinkStrength$***:**        $core\left[v_{i}\right]=1;commNo\left[v_{i}\right]=commNo\left[v\right];$

Community labels of the core vertices around a seed are assigned to the seed’s community label. These community labels then propagate to neighbors of the community cores, so that, at the end, each node is assigned one community label. All vertices with the same community label form a community. The community labels propagate using the majority principle of label propagation. Instead of counting links to different communities, we use the sum of link strengths to different communities. Community label propagation is performed in more iterations until all vertices have their community label. We do not choose unlabeled vertices for label propagation randomly, but we choose the unlabeled vertices with the highest density-based entropy centrality value for the propagation continuously, with the aim to avoid different resulting partitions in multiple runs and to improve the labeling quality in an computationally efficient label propagation.

For listing all the maximal cliques in sparse graphs, the node-ordering version of the Bron–Kerbosch algorithm can be made to run in time $O(dn3^{d}/3)$, where *d* is the degeneracy of the graph and a measure of its sparseness [40]. Computation of the vertices’ density-based entropy centrality requires the neighbors of each node, which can be identified in O(1) time using the adjacency matrix data structure. The time complexity of the calculation of density-based entropy centrality is $O(d^{2})$ (Equation (9)), where *d* is the average node degree (the average number of the node’s neighbors). The time complexity of the calculation of density-based entropy centrality for *n* vertices is O(n). Each node is initialized with a unique node label in O(n) time. The sorting of important values can be performed with the time complexity O(n·log(n)). Then, the link strength is calculated for all edges (Equation (8)). The calculation of the maximal cliques can be computed in polynomial-time [41]. Then, in the second step, the core vertices are identified in O(m) time, where *m* is the number of edges. In the third step, the label propagation of the community central candidates has near linear time complexity O(n). The total time complexity of the proposed algorithm is O(m+n·log(n)).

## 6. Experiments and Analysis

We tested the performance of the proposed density-based entropy centrality measure on real network structures. All the real-world datasets used for testing are listed in Table 1.

The measures that are used most often for the evaluation of community-detection methods are Normalized Mutual Information (NMI) [42] for the evaluation of synthetic datasets with known resulting communities, the modularity *Q* [43] for evaluation of the quality of the communities in the real-world datasets, and the F1-score measure for assessing the performances of a community-detection algorithm for large datasets [44].

**Table 1:** Networks used in the experiments with the number of vertices, edges, and averaged node degrees.

Networks	Vertices	Edges	Average Degree	Description	Reference
Karate	34	78	4.6	Zachary’s Karate Club	[45]
Dolphins	62	159	5.1	Dolphins social network	[46]
Polbooks	105	441	4.2	Books about US politics	[47]
Polblocks	1490	19,062	12.8	Hyperlinks in blogs on US politics	[48]
Football	115	613	10.6	American college football	[49]
Jazz	198	2742	27.7	Jazz musicians network	[50]
Ecoli	423	519	2.4	Biological network	[51]
Power Grid	4941	6594	2.7	The Western States Power Grid in US	[52]
PGP	10,680	24,340	4.5	Yeast PPI dataset	[53]
DBPL	317,080	925,872	5.8	Co-authorship network	[54]
YouTube	1,134,890	2,987,624	5.3	Video-sharing website users	[54]
Amazon	334,863	925,827	5.5	Who-Bought-This-Item-Also-Bought	
				customers feature in Amazon website	[54]

### 6.1. Real-World Networks

We tested the performance of the community detection using a density-based entropy on the twelve real networks listed in Table 1. The testing set of networks consisted of one biological network, one technical network, and ten social networks.

The Zachary karate network contains 34 members of a university karate club. The links model the interaction of the members outside the club. A conflict between an administrator and an instructor led to the split of the club into two clubs.

The Dolphins’ dataset is a network of 62 dolphins living in New Zealand. The nodes in the network represent the dolphins, and the links connect two dolphins with frequent contact. There are two communities of dolphins.

US politics books is a social network of books about US politics. Each node in the network represents a book, and a link between two books indicates that they are often bought together. There are three communities in the network.

US political blogs is a network of Internet blogs on the subject of US politics with partitioning of the graph into liberal and conservative bloggers.

The American College football network models American football games between Division IA colleges during the regular season Fall 2000.

The Jazz network is the collaboration network among Jazz musicians.

We also tested our method on several networks without ground-truth community partitions (see Table 1): the Ecoli network dataset, the Power Grid dataset represents the topology of the Western States Power Grid of the United States, and the PGP network of users using the Pretty-Good-Privacy algorithm for secure information interchange.

We also tested the proposed centrality on some large-scale network datasets from the SNAP datasets [54]: DBLP, YouTube, and Amazon (see Table 1). The DBLP network is a co-authorship network where two authors of computer science papers are connected if they publish at least one paper together. The ground-truth communities are defined by the publication journal or conference because all the authors who published in a certain journal or conference form a community.

YouTube is a popular video-sharing website, where the users can create groups that other users can join. The user-defined groups are the ground-truth communities of the network.

The Amazon network is based on Customers-Who-Bought-This-Item-Also-Bought feature of the Amazon website. An undirected edge between two products denotes that the products are co-purchased together frequently. The product category provided by Amazon defines each ground-truth community.

### 6.2. Experimental Results

**Figure 1:**
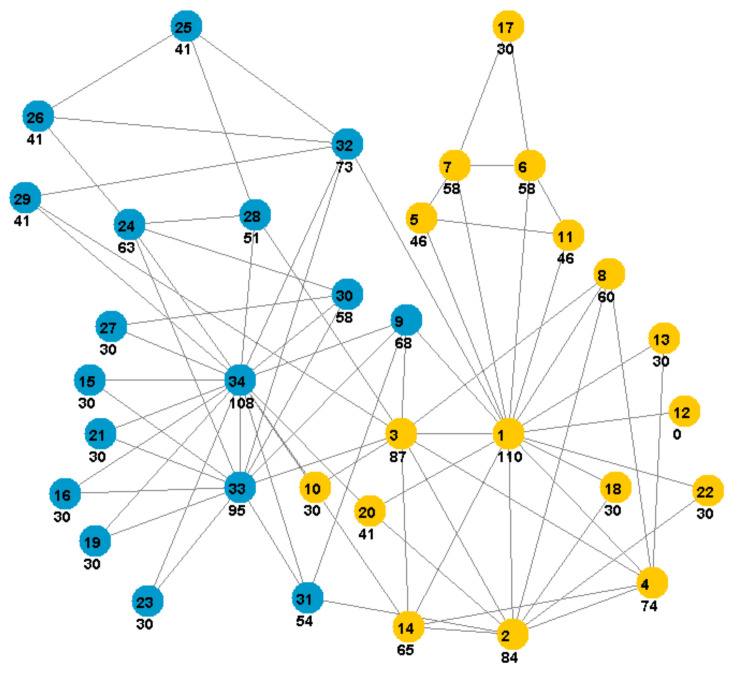
Karate network and two identified communities via CDCE. Vertices with the same color form a community. Numbers in circles are vertices’ labels, followed below by density-based centrality values · 100.

The application of the proposed method to the Zachary karate network is shown in Figure 1. We found that the most central node has a label of 1 (density-based centrality 110) and that the second highest is node 34 (density-based centrality 108) and then node 33 (density-based centrality 95) (see Figure 1). The two identified communities model two groups of members that are in conflict, with the result of splitting the club into two clubs.

**Figure 2:**
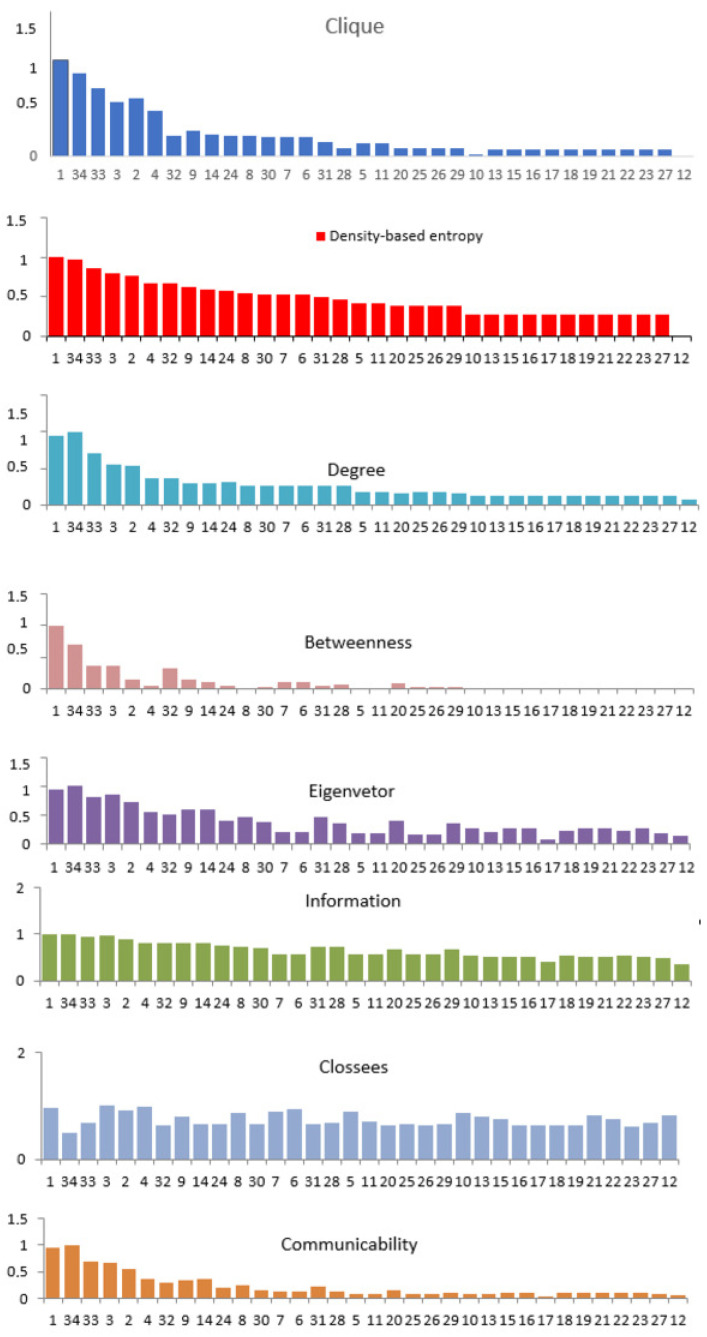
Karate network and node centrality values using different centrality measures: clique centrality, density-based entropy centrality, degree, betweenness, eigenvector, communicability, closeness, and information centrality.

In Figure 2, we note that there is a difference between the ranking of density-based centrality measures of nodes in the Zachary karate club network and the other centrality measures. We emphasize the difference between the results obtained with density-based centrality and those obtained with clique centrality, although both measures have similar heuristics. We can see that the most important vertices (1,34,33) have the highest density-based entropy centrality values. However, other vertices also have higher density-based entropy centrality values than other centrality values, including clique centrality. This also allows for locally identifying the most important nodes in weakly connected parts of network and for forming not only strongly connected communities but also weakly connected communities.

For the dolphin datasets, political books, and political blogs, all the real datasets were uncovered with a resulting modularity better than that of the other considered methods (see Table 2).

**Figure 3:**
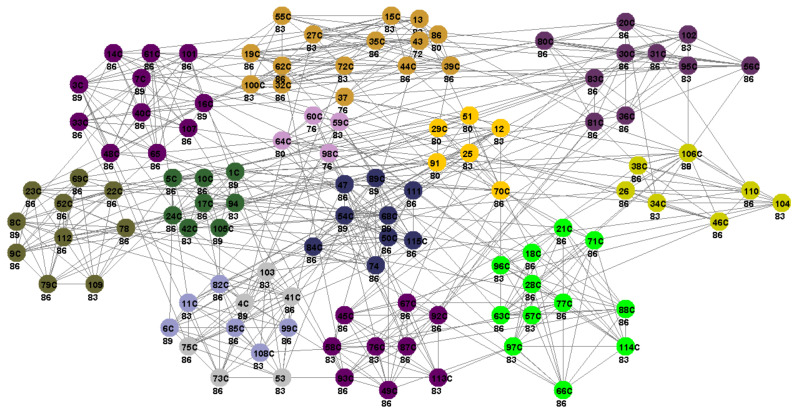
The partition results obtained via the proposed method CDCE for football network. Vertices with the same color form a community. Numbers in circles are vertices’ labels, followed below by density-based centrality values · 100. Core vertices are marked with the character ‘C’.

Community detection of the American football dataset divides the football teams into 12 groups or conferences, with more frequent intra-conference matches than inter-conference matches. All the real communities are identified and shown in Figure 3.

**Figure 4:**
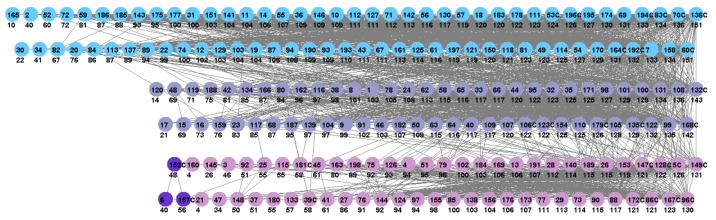
The partition results for jazz network with three overlapped communities uncovered. Vertices with the same color form a community. Numbers in circles are vertices’ labels, followed below by density-based centrality values · 100. Core vertices are marked with the character ‘C’.

The Jazz network is separated into three real communities, and all three identified are shown in Figure 4, where two overlapping communities (blue and violet) are connected very densely and, thus, were difficult to uncover.

The results of Jazz communities prove that the proposed method and the use of density-based entropy centrality enables the efficient identification of also overlapping communities.

The improved label propagation method CDCE using the proposed centrality uncovered all the real communities (also very overlapping) in the upper real-world datasets often used as a test-bed for the evaluation of community-detection methods. We also evaluated the efficiency on the large datasets below.

For assessing the performances of a community-detection algorithm for large datasets, we used the F1-score measure proposed by Rossetti et al. [44]. The F1-measure obtained with the proposed method compared with the values published by Rossetti et al. also showed the efficiency of the proposed method for identifying the communities also in large real-world networks (see Table 3).

From the upper F1-scores, we can see that the use of the proposed centrality is efficient, although cliques larger in size than four tend to be very sparse in large networks. The proposed density-based entropy centrality emphasizes the power of the maximal cliques in defining the central node and in community detection. The vertices in each community tend to be interconnected densely and may form multiple cliques with large sizes. We considered the sum of all the maximal sizes of these cliques and not only above some certain threshold. The maximal cliques allowed for encoding all clique information adaptively, based on whatever clique sizes are available.

We compared the results of CDCE using different centrality measures for small real-world datasets often used as a test-bed for the evaluation of community-detection methods. We used the density-based entropy centrality, clique centrality and degree centrality. Even for these small datasets, the density-based centrality performed better than degree and graph centrality, as shown in Table 4. It can be seen that the best results were obtained using the density-based entropy centrality. Degree centrality can sometimes identify vertices that are bridges between two or more communities, instead of the real centers of communities. Identification of the wrong centers can lead to the identification of unreal communities. Using degree centrality gives communities with the smallest modularity values for the most considered dataset (see Table 4). Using graph centrality identifies centers, which give, as a result, communities with smaller modularity (for four datasets from six) than using density-based entropy centrality.

**Figure 5:**
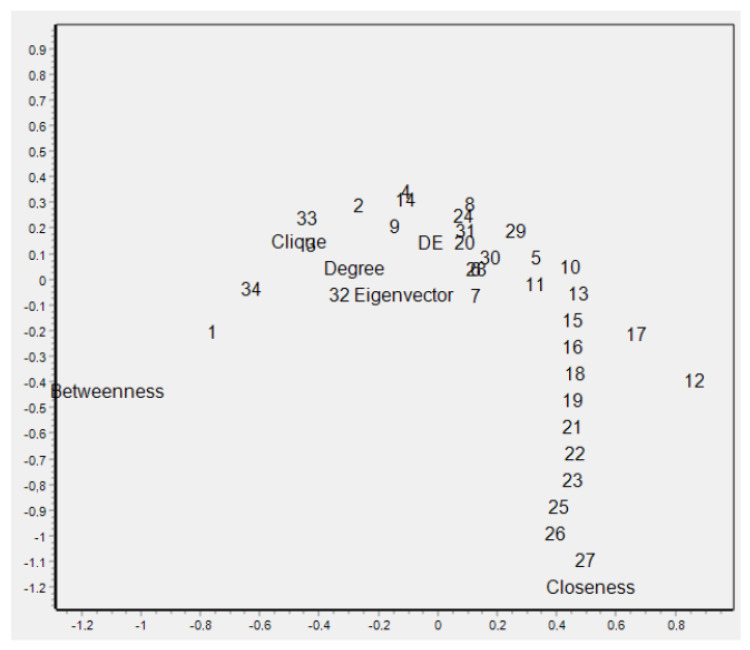
Correspondence analysis for centrality measures for karate club data.

The correlation of different centrality measures is shown in the correspondence graph in Figure 5. Clique centrality is labeled “Clique”, density-based entropy centrality is labeled as “DE”, and the members of the karate club are labeled with numbers 1 through 34. In Figure 5, clique centrality is a central measure, correlating with degree centrality. We demonstrate that the proposed centrality measure is correlated with local centrality (degree centrality) and correlated weakly with the global node centrality measures. The local nature of density-based entropy centrality is useful for identification of the most important vertices, which are the seeds of communities, while the communities are local structures.

### 6.3. Comparison with Other Methods

We tested our proposed method on real-world network datasets. We compared the obtained results with the results published for the popular Louvain algorithm [55], which optimizes the modularity measure; Infomap [56], since it is one of the best-performing methods; and LPA [10]. Infomap focuses on trying to compress the list of vertices visited by a random walker on a graph, with the aim to obtain a description of the random walk, which is as short as possible. From Table 2, we can see that, for small networks with ground-truth communities, our method often performs better than other algorithms. For the real-world networks without the ground-truth information (*Ecoli*, Power Grid, and the PGP dataset), we can see that the modularity values of our partitions are lower than those obtained with the Louvain, as CDCE does not optimize modularity as the Louvain algorithm does. However, the modularity values obtained via our method were almost always equal or better than those of the other two algorithms, i.e., Infomap and Label propagation (see Table 2). The results in Table 2 show that CDCE is competitive in most of the considered networks, with the other considered algorithms used for community detection. The results of the CDCE method using only the seeds of communities without core detection show lower modularity than those obtained with the whole proposed CDCE algorithm.

CDCE is particularly efficient for networks whose community centers have sparse inter-connections between each other (e.g., PGP and Power Grid) and also for overlapping communities (e.g., Jazz).

For large datasets, the F1-measure obtained with the proposed method and compared with values published by Rossetti et al. [44] also shows the efficiency of the proposed method for identifying communities also in large real-world networks (see Table 3). For the Amazon and DBLP datasets, the highest F1-score was obtained using the proposed method, while for the YouTube dataset, the same F1-score was obtained as that obtained using the Louvain method.

## 7. Conclusions

This article introduces density-based entropy centrality. It is a local measure of node centrality. The proposed density-based entropy centrality can be applied efficiently for community detection, which is also efficient for the identification of dense and overlapping communities. We identified the seed vertices, and then, the extended LPA method was used to identify the final communities. The empirical evaluations on real-world networks show that the used method identifies more ground-truth community members more efficiently than the other considered methods. The use of density-based entropy centrality gave better results than two other considered local centrality measures: clique centrality and degree centrality.

The use of the centrality measure and a community-detection method for identifying communities in specific real-world networks like Facebook can be our future research work. The enhancement of a method for identifying dynamic communities can be a promising direction of our research.

## Figures and Tables

**Table 2:** The results shown here are the modularity (Q) obtained via the CDCE and the considered methods (Infomap, LPA, and Louvian) and the number of uncovered communities when different from the real number in brackets.

	CDCE without Core Detection	CDCE	Infomap	LPA	Louvian
Karate	0.372	0.372	0.4	0.37	0.42
Dolphin	0.490	**0.527**	0.52	0.5	0.52
Polbooks	0.457	**0.52**	0.52	0.5	0.52
Jazz	0.021	**0.44**	0.28 (7)	0.28 (2)	0.44 (4)
Polblogs	0.426	**0.43**	0.42	0.43	0.43
Football	0.57	0.577	0.6	0.57	0.6
Ecoli	0.717 (67)	0.717 (67)	0.71 (39)	0.68 (42)	0.88 (102)
PowerGrid	0.75 (586)	0.767 (563)	0.82 (483)	0.81 (479)	0.93 (40)
PGP	0.81 (960)	0.84 (357)	0.82 (1070)	0.81 (955)	0.88 (190)

**Table 3:** The F1-score obtained via the CDCE, Louvain, and Infomap for Amazon, DBPL, and YouTube datasets.

Dataset/Method	CDCE	Louvain	Infomap
Amazon	0.463	0.40	0.46
DBLP	0.57	0.26	0.45
YouTube	0.16	0.16	0.59

**Table 4:** The results shown here are the modularity (Q) obtained via the CDCE using density-based entropy centrality, clique centrality, and degree centrality.

	CDCE Using Density-Based Entropy Centrality	CDCE Using Clique Centrality	CDCE Using Degree Centrality
Karate	**0.372**	0.371	0.37
Dolphin	**0.527**	0.52	0.5
Polbooks	0.52	0.52	0.52
Jazz	**0.44**	0.439	0.439
Polblogs	**0.43**	0.426	0.425
Football	**0.577**	**0.577**	0.553

## Data Availability

Not applicable.
